# Comprehensive Physiotherapy Rehabilitation Protocol of Plantar Fasciitis for a 45-Year-Old Female: A Case Report

**DOI:** 10.7759/cureus.51585

**Published:** 2024-01-03

**Authors:** Manali A Boob, Pratik Phansopkar, Kamya J Somaiya

**Affiliations:** 1 Musculoskeletal Physiotherapy, Ravi Nair Physiotherapy College, Datta Meghe Institute of Higher Education and Research, Wardha, IND

**Keywords:** rehabilitation training, plantar heel pain, foot functional index, windlass test, plantar fasciitis

## Abstract

Plantar fasciitis is stated to arise because of inadequate accumulated tension at the plantar fascia's enthesis. Tensile load and prolonged strain cause tiny rips in the fascia, which trigger a chronic inflammation process of healing. This case report shows the diagnostic evaluations, assessment of the condition, and physical rehabilitation management for a 45-year-old female nurse working in the neurosurgical critical care unit who had been experiencing plantar medial and posterior heel pain, as well as discomfort at the calcaneal tuberosity, for the previous six months. To increase functional mobility and alleviate symptoms, the patient sought out physiotherapy intervention. In this case, a physiotherapeutic program was implemented to treat plantar fasciitis, enhance mobility, and encourage long-term recovery. The evaluation included a detailed review of the patient's gait, biomechanics, and circumstances that may have contributed to the ongoing problems. The multimodal strategy used in the intervention plan included manual therapy, strengthening and stretching exercises, as well as patient education and counselling on self-management techniques. The patient's functional mobility increased along with a steady reduction in discomfort during the duration of the physiotherapy sessions. The instance emphasises how important it is to manage persistent plantar fasciitis with a customised physical therapy strategy that takes the patient's specific requirements into account and addresses contributory variables. The present study adds to the extant literature on efficacious physiotherapeutic approaches for plantar fasciitis, highlighting the need for a holistic approach in attaining favourable results for individuals enduring heel discomfort.

## Introduction

Fascia is a type of connective tissue with three layers that vary with the structures around it. It is interconnected in bands as one unit, delivering tensile properties to the body [1]. Plantar fasciitis is hypothesised to be caused by excessive accumulated tension in the fascia. The mechanical stress and severe tension cause tiny breaks in the fascia, which set off an inflammatory reaction for healing [2]. Foot pain in plantar fasciitis presents as morning stiffness and plantar area pain [3]. The cause of this illness is currently unknown. Several studies suggest a link between the weakening of the intrinsic muscles of the foot and the various abnormalities that affect the foot arch, such as pes cavus and pes planus [4]. Ultrasound analysis suggests fibrosis, thickening, collagen breakage, hypertrophy and necrosis of the plantar fascia [5]. Plantar fasciitis is more frequent between the ages of 40 and 60. Nonoperative treatment relieves symptoms in more than 80% of individuals [6].

The foot functional index was designed to evaluate how foot pathology affects pain, disability, and capacity for certain activities. The most popular foot-specific self-reporting tool is the foot functional index, which is also a frequently used outcome measure for assessing plantar fasciitis patients. In several populations with a range of foot and ankle conditions, the foot functional index has proven to be valid, dependable, and responsive to change. Subscale scores range from 0% to 100%, with higher scores indicating lower levels of function and poorer foot health-related quality of life [7]. According to clinical practice guidelines, there is diverse empirical evidence to support the effectiveness of various interventions for treating plantar fasciitis. Plantar fasciitis is nowadays treated with a wide variety of traditional methods, such as splints, oral and injectable anti-inflammatory drugs, therapeutic taping, manual therapy, foot orthoses, calf stretching, etc [8]. This case study describes a female nurse who was diagnosed with plantar fasciitis and underwent rehabilitation. After the intervention, the patient's symptoms were relieved, and she was able to carry out everyday duties with ease.

## Case presentation

**Figure 1:**
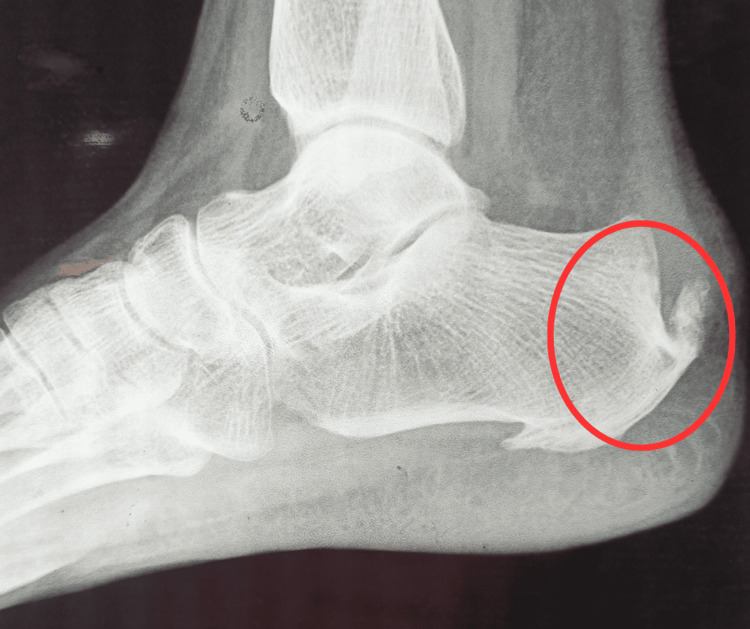
Radiographic representation of the lateral view of the right-side ankle joint, which shows calcaneal spur.

**Figure 2:**
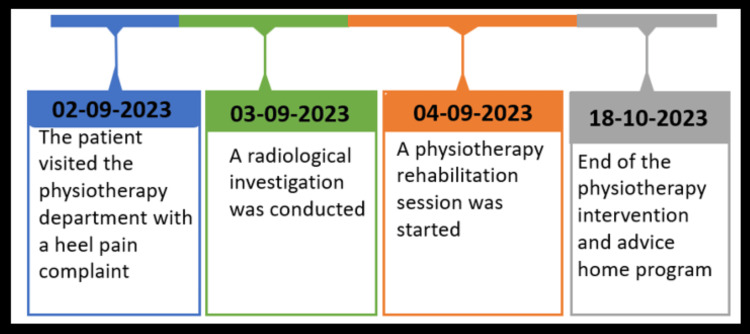
Timeline of the events.

A 45-year-old female nurse who was working in the neurosurgery ICU and had a body mass index of 21.2 kilograms per square meter came to the physiotherapy department. The female had complained of pain at the plantar medial aspect of the heel and posterior part of the heel since the sixth month. This pain was sharp and most noticeable in the first few steps of the morning. It subsided after thirty minutes of walking. At rest, there was no pain. The patient was having difficulty walking and maintaining balance due to pain and dorsiflexor weakness for one month. She gave a history of seven to eight hours of prolonged standing per day. The patient had a history of diabetes mellitus for the past two years. Before the patient was evaluated, their oral and written informed consent was obtained. Heel pain was rated on the numerical pain rating scale during the assessment, which was mentioned in the outcome section. The squeeze test for calcaneal stress fracture and the Tinel sign for tarsal tunnel syndrome were both negative. There was swelling and grade two tenderness at the right heel, and the Achilles tendon showed tightness. The windlass test was positive. The dorsiflexion range of the right side was reduced compared to the left side. There was no difference in limb length. Manual muscle testing was mentioned in the outcome measure section. The strength of the dorsiflexors on the right side was reduced. The radiograph showed the formation of a heel spur (Figure 1). From the above positive finding, the patient was suffering from plantar fasciitis. The timeline of the event is mentioned in Figure 2.

Therapeutic intervention

A rehabilitation training regime seeks to relieve discomfort, improve mobility, and promote long-term recovery. Through focused stretching and strengthening exercises, the major aims include reducing inflammation and stress in the plantar fascia. Physiotherapy seeks to increase the flexibility of the calf muscles and Achilles tendon while treating the underlying causes of plantar fasciitis. For the calcaneal spur, cortisone injections were given twice, botulinum injection once and the same rehabilitation program was given as that of plantar fasciitis. Furthermore, the regimen focuses on improving proprioception, balance, and stability to reduce the chance of re-injury and promote overall foot care. Table 1 displays the therapeutic intervention.

**Table 1:** Detailed therapeutic intervention according to the patient's problem

Goals	Therapeutic Interventions
Patient education for the condition and the intervention	Give the importance of compliance with the prescribed interventions. Emphasise the role of lifestyle modifications in preventing future episodes.
To alleviate and prevent plantar fasciitis pain, enhancing patients’ mobility and overall well-being.	Rest: Giving your feet adequate rest is important. Avoid activities that exacerbate the pain and try to minimise weight-bearing on the affected foot.
	Ice: Apply ice to the painful site for 15-20 minutes. Advise to do it several times a day.
	Ultrasound (Figure 3): At the medial calcaneus tuberosity with eight minutes of duration with a frequency of 1 megahertz and therapeutic intensity of 1.8 watts per square centimetre.
	Stretching exercises: Specific stretching helps to alleviate tension on the plantar fascia, which includes calf stretches, Achilles tendon stretches, etc.
	Orthotics: Custom or over-the-counter shoe inserts (orthotics) can provide support. They can improve foot alignment, and kinesio taping (Figure 4) was done to decrease strain and pain on the plantar fascia.
To improve the range of motion and flexibility of the dorsiflexors	Calf stretch: Put hands against a wall while facing it. Keep one foot back with a straight knee, and bend the front knee. Lean forward on the wall while keeping the back heel on the ground. Maintain position for 15 to 30 seconds and relax. Repeat two or three sets on each day.
	Towel stretch: Anchor a towel around the ball of the foot. Gently pull the towel and hold the stretch for the next 30 seconds, and repeat on each foot.
	Plantar fascia stretch: With one limb placed over the other knee, take a seat in a chair. To stretch the plantar fascia, gradually bring the toes back towards the shin of the tibia. After 30 seconds of stretch, switch to the other foot.
	Eccentric calf raises: Raise your toes with both feet. Lower one foot slowly while keeping the other foot elevated. Then, push up with both feet again. Ten to fifteen sets per day. Ankle range of motion exercises to increase dorsiflexion.
To improve the strength of the plantar and dorsiflexors	Intrinsic muscle strengthening: Sit or stand and try to lift the arches of your feet, spreading your toes apart, performing ball roll (Figure 5A) and towel curls (Figure 5B). Perform two to three sets of ten repetitions.
	Resistance band exercises: Loop a resistance band around the foot to improve plantar flexors and to improve the dorsiflexor's strength and resistance apply in the opposite direction. Perform ten to fifteen repetitions.
To improve balance and stability	Balance exercises: Practice standing on one leg to improve balance and stability. Ankle proprioceptive neuromuscular facilitation for plantarflexion and inversion are shown in Figure 6A. Dorsiflexion and eversion are shown in Figure 6B.
	Proprioceptive training: A wobbleboard (Figure 7A) creates an unstable surface, forcing the foot and ankle muscles to work harder to maintain balance. Bilateral calf raises (Figure 7B) help to improve balance and stability.
	Incorporating uneven surfaces: Walking or performing exercises on uneven surfaces, such as grass or sand, can challenge the proprioceptive system and improve stability.
	Alphabet exercises: Draw the alphabet with toes in the air to promote ankle mobility and strengthen the muscles around the ankle joint.
	Joint position sense training: Perform exercises that focus on joint position sense, such as standing with eyes closed to rely more on proprioception.
	Progressive loading: Gradually increase the difficulty and intensity of proprioceptive exercises as the strength and stability improve.

**Figure 3:**
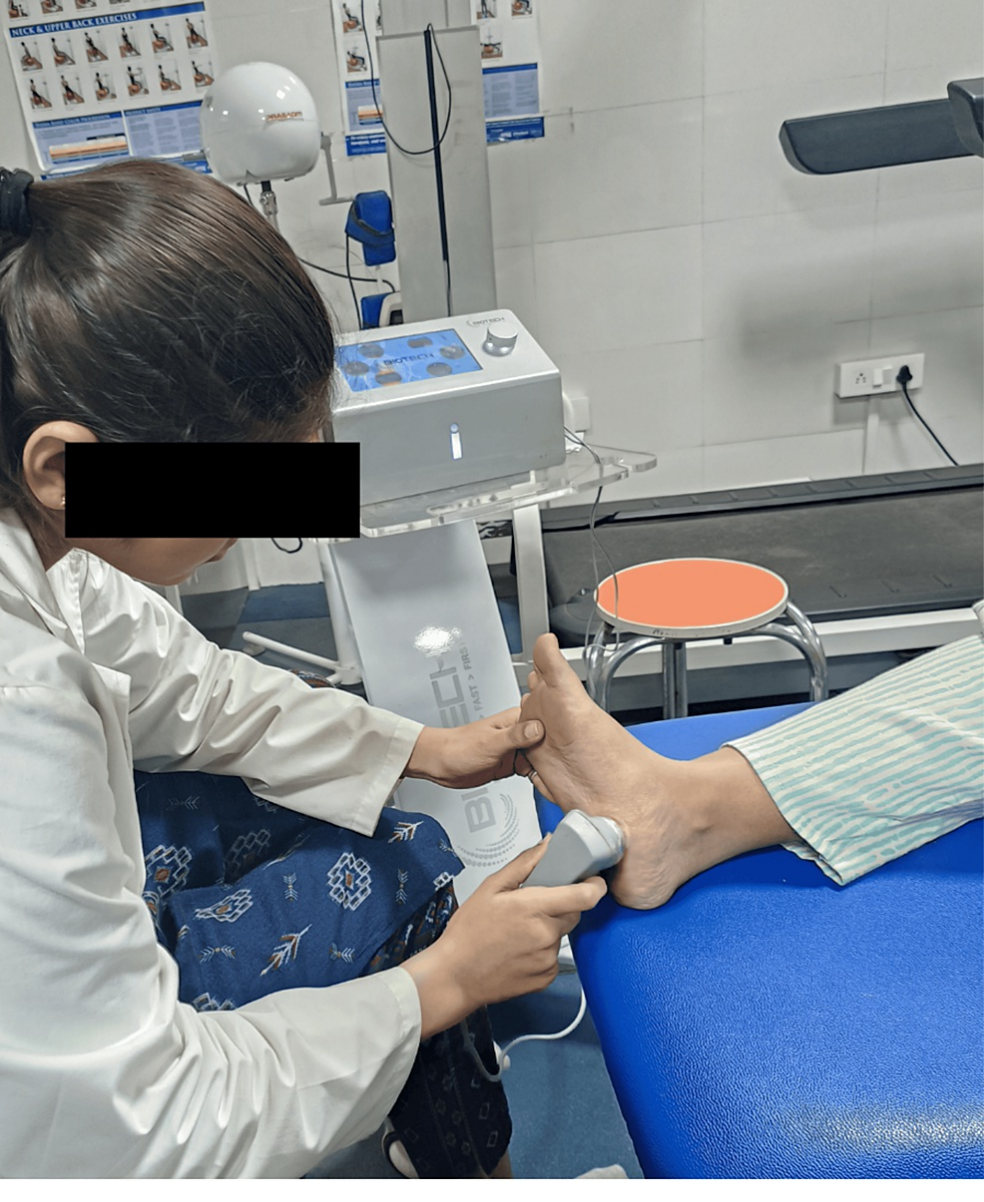
The treatment of plantar fasciitis pain using ultrasound with a frequency of 1 megahertz and therapeutic intensity of 1.8 watts per square centimetre.

**Figure 4:**
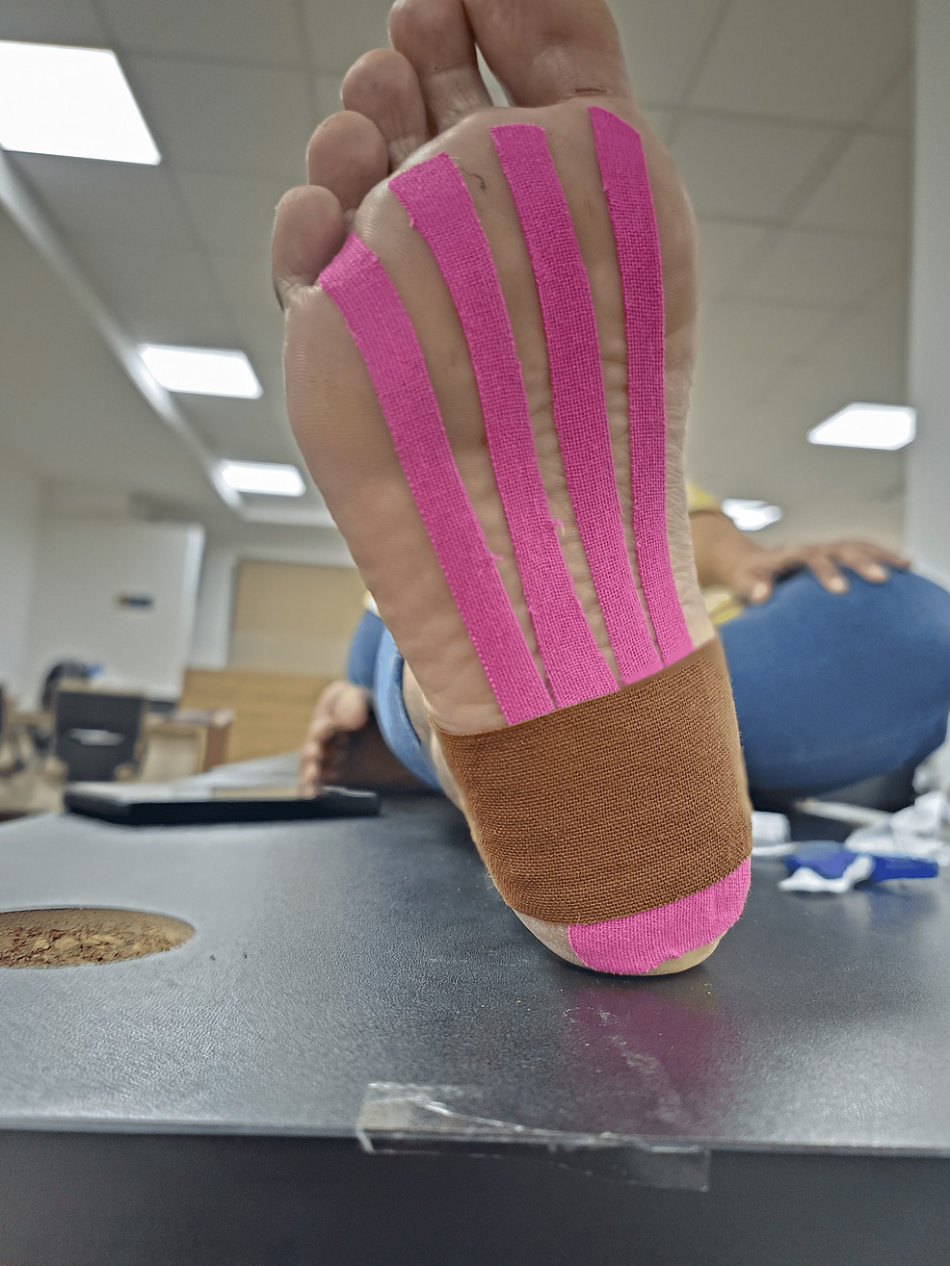
Kinesio taping for plantar fasciitis

**Figure 5:**
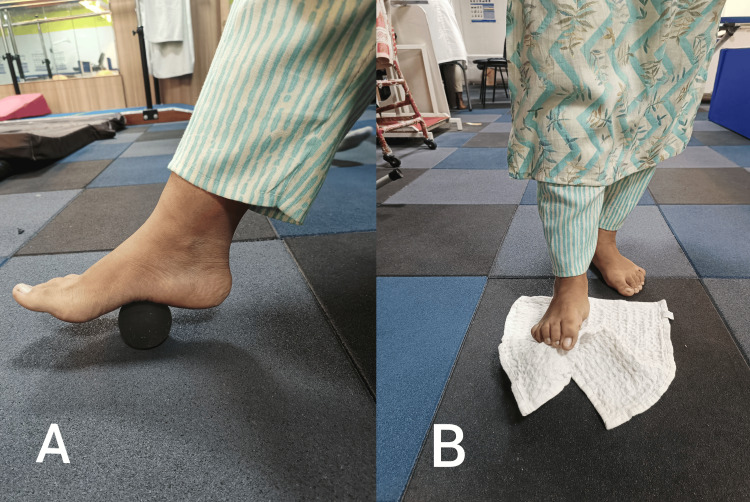
The strengthening of the intrinsic muscles of the foot. A: Ball roll exercise, B: Towel curls exercise

**Figure 6:**
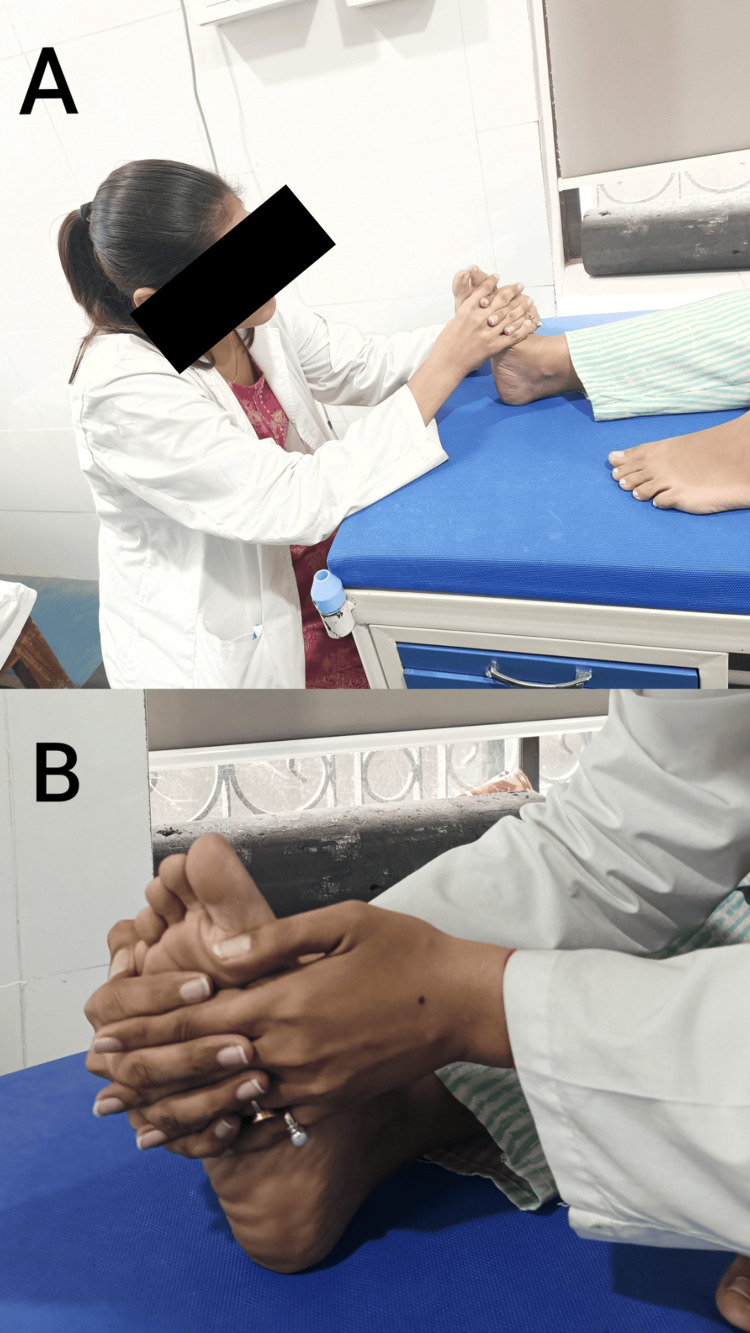
The ankle proprioceptive neuromuscular facilitation to increase balance. A: Plantarflexion and Inversion, B: Dorsiflexion and Eversion

**Figure 7:**
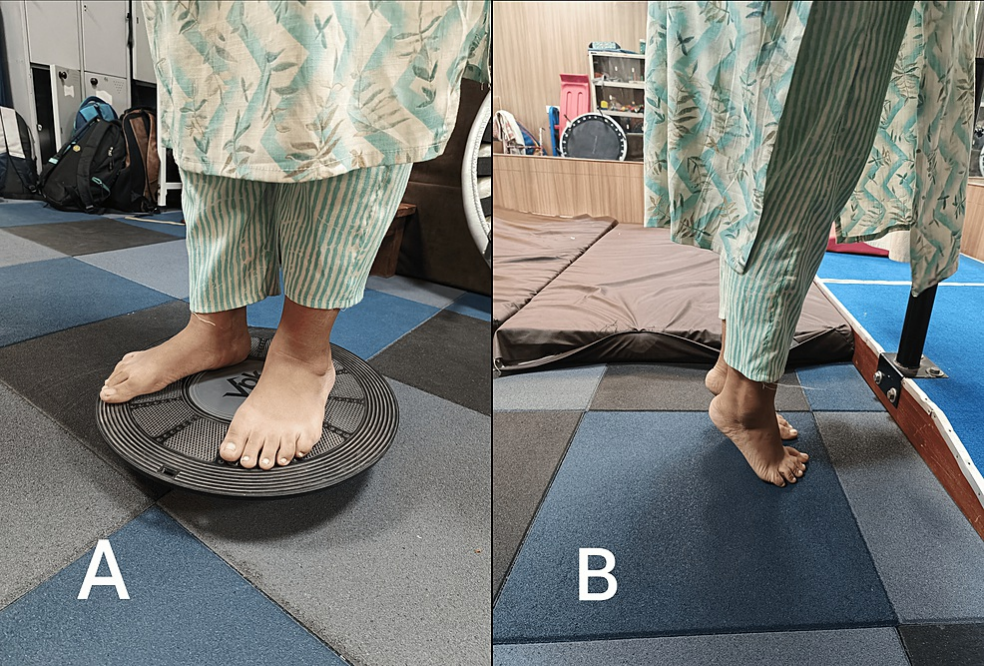
The proprioceptive training. A: Balance board standing, B: Bilateral calf raises

Outcome measure

Pre-physiotherapy outcomes were measured on the first day before the treatment was initiated and post-physiotherapy outcome measures were measured after the four weeks of the physiotherapy intervention. To evaluate the patient's stated pain, the visual analogue scale was used. By using the goniometer, the range of motion was assessed.

The manual muscle test was used to assess muscular strength. In order to rate and evaluate the status of the foot, the foot functional index was used. The study highlights the significant improvement in the outcome measures after the physiotherapy regimen. The outcome measures are mentioned in Table 2.

**Table 2:** The pre- and post-rehabilitation outcome measures NPRS: numerical pain rating scale; FFI: foot functional index; ROM: range of motion; MMT: manual muscle testing Numerical pain rating scale interpretation: (0) - no pain, (1-3) - mild pain, (4-5) - moderate pain, (6-7) - severe pain, (8-9) - very severe pain, (10) - worst pain Manual muscle testing interpretation: 0 - no muscle contraction, 1 - flickering muscle contraction, 2 - full range of motion with gravity eliminated plane, 3 - full range of motion against gravity plane, 4 - full range of motion against gravity plane with minimal resistance, 5 - full range of motion against gravity plane with minimal resistance

Parameters	Pre-physiotherapy Intervention	Post-physiotherapy Intervention
Numerical pain rating scale (NPRS)	Seven out of ten	Three out of ten
Foot functional index (FFI)	60 percent	20 percent
Plantarflexion (ROM)	50 degrees	50 degrees
Dorsiflexion (ROM)	8 degrees	20 degrees
Plantarflexor (MMT)	5/5	5/5
Dorsiflexors (MMT)	3/5	5/5

## Discussion

Plantar fasciitis is termed an inflammation of the plantar fascia. The issue is often caused by repeated stress, overuse, or incorrect foot mechanics, which results in microtears and fascia disruption. This deterioration causes discomfort, stiffness, and sensitivity, especially around the heel. Plantar fasciitis is frequently accompanied by the development of a calcaneal spur, a bony protrusion on the underside of the heel bone. In sports medicine, plantar fasciitis and the accompanying heel discomfort are prevalent ailments. While there is a mostly consistent pathophysiological concept, a wide range of therapy approaches have been suggested to treat plantar fasciitis [2]. It hinders regular activities and creates substantial pain and incapacity for the sufferer. Plantar fasciitis is caused by unsuitable footwear, which has a small heel, thin soles, and firm insoles with no built-in arch support [9]. During walking, the plantar fascia's orientation is crucial for keeping the arches in alignment. Different phases of the gait cycle are affected by inefficient foot mechanics. Both the plantar fascia and the calcaneal tubercle get irritated by prolonged tension. An overabundance of pronation may cause the plantar fascia to extend and the tibialis posterior to weaken. The lengthening prevents the best possible use of the foot's windlass mechanism due to the imbalance experienced during the propelling phase of ambulation. The tibialis posterior and intrinsic muscles can be strengthened to regain this function.

Poor foot posture puts an excessive amount of stress on the lower extremity muscles and bones, which can lead to anatomical alterations throughout the posture [10]. Kaur and Koley compared Achilles and calf stretching in plantar fasciitis patients and suggested that Achilles tendon stretching for four weeks causes significant symptom relief [11]. Cohena-Jimenez et al. stated that the use of foot orthosis had an excellent result, which helps to improve stability and reduce discomfort. It is beneficial for long-term benefits [12]. Yadav et al. evaluated the impact of foam rolling in addition to self-stretching versus self-stretching alone on plantar fasciitis patients. The results of the study showed that self-stretching and foam rolling can help individuals with plantar fasciitis reduce discomfort and improve their range of motion [13]. Nadeem et al. stated that the ergon technique is a remarkable physiotherapeutic intervention to improve strength and joint mobility [14]. The therapeutic intervention in this study such as ankle proprioception facilitation and the use of the kineso taping demonstrates a noteworthy improvement in balance and the stability of the patient's daily living activities.

## Conclusions

In conclusion, the successful alleviation of plantar fasciitis symptoms in a 45-year-old female nurse with physiotherapeutic intervention highlights the efficacy of non-invasive rehabilitation in managing this condition. Her functional mobility was generally improved by the all-encompassing physiotherapy approach, which included focused exercises, stretching exercises, and manual treatments in addition to relieving the acute discomfort associated with plantar fasciitis. This example emphasises the critical role that physiotherapists play in improving the quality of life for people dealing with musculoskeletal problems and summarises the significance of customised rehabilitation programmes catered to the unique requirements of patients. Rehabilitation is a cornerstone in the holistic care and management of conditions like plantar fasciitis, promoting recovery and restoring patients to a state of optimal health.
